# Stochastic Recognition of Human Physical Activities via Augmented Feature Descriptors and Random Forest Model

**DOI:** 10.3390/s22176632

**Published:** 2022-09-02

**Authors:** Sheikh Badar ud din Tahir, Abdul Basit Dogar, Rubia Fatima, Affan Yasin, Muhammad Shafiq, Javed Ali Khan, Muhammad Assam, Abdullah Mohamed, El-Awady Attia

**Affiliations:** 1 Department of Software Engineering, Capital University of Science and Technology (CUST), Islamabad 44000, Pakistan; 2 Department of Computer Science and Technology, Tsinghua University, Beijing 100084, China; 3 School of Software Engineering, Tsinghua University, Beijing 100084, China; 4 School of Information Engineering, Qujing Normal University, Qujing 655011, China; 5 Department of Software Engineering, University of Science and Technology, Bannu 28100, Pakistan; 6 Research Centre, Future University in Egypt, New Cairo 11745, Egypt; 7 Department of Industrial Engineering, College of Engineering, Prince Sattam Bin Abdulaziz University, Al Kharj 16273, Saudi Arabia; 8 Mechanical Engineering Department, Faculty of Engineering (Shoubra), Benha University, Cairo 11629, Egypt

**Keywords:** human physical activity recognition (HPAR), Hilbert–Huang transform (HHT), inertial measurement unit (IMU), stochastic gradient descent (SGD)

## Abstract

Human physical activity recognition from inertial sensors is shown to be a successful approach for monitoring elderly individuals and children in indoor and outdoor environments. As a result, researchers have shown significant interest in developing state-of-the-art machine learning methods capable of utilizing inertial sensor data and providing key decision support in different scenarios. This paper analyzes data-driven techniques for recognizing human daily living activities. Therefore, to improve the recognition and classification of human physical activities (for example, walking, drinking, and running), we introduced a model that integrates data preprocessing methods (such as denoising) along with major domain features (such as time, frequency, wavelet, and time–frequency features). Following that, stochastic gradient descent (SGD) is used to improve the performance of the extracted features. The selected features are catered to the random forest classifier to detect and monitor human physical activities. Additionally, the proposed HPAR system was evaluated on five benchmark datasets, namely the IM-WSHA, PAMAP-2, UCI HAR, MobiAct, and MOTIONSENSE databases. The experimental results show that the HPAR system outperformed the present state-of-the-art methods with recognition rates of 90.18%, 91.25%, 91.83%, 90.46%, and 92.16% from the IM-WSHA, PAMAP-2, UCI HAR, MobiAct, and MOTIONSENSE datasets, respectively. The proposed HPAR model has potential applications in healthcare, gaming, smart homes, security, and surveillance.

## 1. Introduction

Human physical activity recognition (HPAR) is a subject of research that focuses on developing and experimenting with novel techniques for automatically recognizing activities via signals acquired by wearable or ambient sensors [1]. However, for the most part, ambient sensors require installation in a household environment, and appliances such as camera systems are seen as obtrusive, specifically by aging people [2]. For such reasons, the emphasis has turned to the employment of wearable sensors in recent years. Fitness trackers, smartphones, and inertial sensors are currently receiving adequate attention [3,4,5,6]. This is mainly due to the widespread use of gadgets by the general public and the incorporation of different types of inertial sensors embedded in electronic devices such as accelerometers, gyroscopes, magnetometers, and GPS.

Human physical activity recognition (HPAR) from wearable body sensor networks is gaining rapid growth due to its significant potential in various application domains, including smart healthcare, smart homes, security, biofeedback systems, assistive robots, and transportation. Each application demands continuous real-time detection and tracking [7,8,9,10,11]. Additionally, these applications could provide significant access to the medical information of unwell seniors. In smart healthcare, critical information about elderly individuals is acquired. That acquired information is sent in real-time to virtual assistant services via communication technologies [12]. Furthermore, these wearable sensors can be exploited to detect and track various elements of human motion. Real-time monitoring and surveillance of the physical movements of elderly individuals and children can help them to feel more secure and confident. In the context of smart homes, in the last decade, smart home technology has shifted its focus towards individuals with limited capabilities (e.g., due to aging or disabilities). This interest is due to the possibility of smart homes assisting elderly adults and individuals with disabilities in their residences while minimizing the significant work required of households or professional caretakers. Similarly, biofeedback treatment is effective when used in conjunction with virtual reality (VR) systems to detect changes in biological functions of the body, such as blood pressure, pulse rate, and heart rate; these systems may also be effective for stress management approaches [13]. By keeping an eye out for suspicious behavior on the part of individuals and anticipating aggressive behavior in advance, unpleasant consequences can be mitigated. For decades, security individuals have been relatively adept at locating suspected individuals. However, humans are fallible and may make false allegations. This is why automated security and surveillance systems have exponentially been gaining attention, (i.e., to assist the monitoring process). Substantial increased criminal activity on a global scale demands the development of more automated state-of-the-art technologies to improve surveillance and produce more precise results [14]. In general, the primary objectives of body-worn-based inertial sensors are to bring effective real-time monitoring and correct the detections of actions, behaviors, and their impacts using sensor data.

Recently, there has been a significant demand for wearable-based inertial sensors for various applications. These novel developments have impacted numerous facets of human life, most notably smart healthcare and daily living monitoring. Our research focuses primarily on inertial sensors, such as accelerometers, gyroscopes, and magnetometers, which allow us to analyze daily human life in various scenarios to track and detect changes in position, body motion, and three-dimensional (3D) spaces [15]. Furthermore, the healthcare sector exploits these sensors to measure physical and emotional activities. However, they can also be utilized to track radical shifts in the user’s position, such as tripping; this data can be used to assist in preventing falls and providing immediate support, mainly to the elderly. Considering the practicality of such wearable inertial sensors, significant barriers remain, such as real-time tracking of the information collected from the sensors on the network. These types of data are generally hard to manage in real time.

In this paper, we propose a human physical activity recognition (HPAR) framework that is intended to lessen the challenges associated with recognizing the daily physical activities of humans by exploiting wearable IMU sensors. Our HPAR framework involves five major steps: data acquisition, a signal filtering process, augmented feature descriptors, feature selection, and recognition. First, we acquired IMU data from five main datasets—IM-WSHA, PAMAP-2, UCI HAR, MobiAct, and MOTIONSENSE. The required input data were passed through numerous denoising methods in which we utilized a median filter for inertial sensor-based benchmark datasets to reduce noise from the original signal. After denoising the inertial signal data, discriminative feature descriptors were extracted from four main domains: time, wavelet, frequency, and time–frequency domain. Additionally, we analyzed and evaluated the feature selection method in conjunction with a state-of-the-art random forest model in order to create a precise model with a compact and discriminative feature vector space. To assess the performance of our HPAR model, we applied our proposed framework to five benchmark datasets: IM-WSHA, PAMAP-2, UCI HAR, MobiAct, and MOTIONSENSE. The results revealed that the proposed HPAR model outperformed other existing state-of-the-art systems. The following are the main contributions and highlights of this paper:

- The augmentation of discriminative features from various domains makes the proposed human physical activity recognition model robust in the presence of noisy data. It maintains locally dependent characteristics of the random forest algorithm, providing a novel approach for improving recognition performances across all five benchmark datasets.
- A hybrid feature descriptor model with random forest is proposed to cope with the convoluted patterns of human physical motion activities with improved classification accuracies in all datasets.
- The complex behavior transition, especially in our self-annotated dataset IM-WSHA, requires more time to recognize activities. Therefore, we utilized a higher window size so that our model could work with a minimal number of changes. We also created a self-annotated dataset named Intelligent Media Wearable Smart Home Activities (IM-WSHA), comprising 11 (static and dynamic) daily life log activities, along with divergences in gender, weight, height, and age.
- Additionally, a comprehensive analysis was performed for human physical activities on five public benchmark datasets: IM-WSHA, PAMAP-2, UCI HAR, MobiAct, and MOTIONSENSE. Experimental results reveal an improved recognition rate, which also outperforms other state-of-the-art systems.

The remainder of this study is structured as follows: Section 2 provides a detailed overview of the literature concerning human physical activity analyses. Section 3 addresses the proposed framework of our HPAR model. Section 4 analyzes the five benchmark datasets along with the detailed experimental results. Finally, Section 5 presents the paper’s conclusion and future research perspectives.

## 2. Related Work

There are two standard ways to analyze HPAR: vision sensor-based HPAR and wearable sensor-based HPAR. Various characteristics and insights may be drawn from this analysis, including the acquired image and signals, extracted feature descriptors, and methods utilized for dimensionality reduction and human activity classification. This section summarizes previous research on human physical activity recognition (HPAR) analyses via vision sensors and wearable sensors.

### 2.1. HPAR via Vision Sensors

Vision-based HPAR relies entirely on visual sensing technologies, including surveillance and video cameras, image sequences, video sequences, modeling, segmentation, detection, and tracking. Liu et al. [16] presented a human activity recognition system incorporating a non-linear support vector machine (SVM) to recognize twenty distinct human activities via an accelerometer and RGBD camera sensor data. Their experimental results indicate that their proposed method is significantly more robust and effective than the baseline method at recognizing activities. However, the main constraint of this study involved the performance of unusual classes, particularly transition activity classes. Additionally, they intended to improve the performance by incorporating this class imbalance issue into their classification model. Yang et al. [17] developed a novel model for identifying human activities from a video series recorded by depth-based cameras. Additionally, they discussed the low-level polynomial designed from a nearby local hyperspace. Furthermore, their proposed system is adaptable, i.e., it could be used in cooperation with the joint trajectory-matched depth sequence. Their proposed model was comprehensively analyzed and tested on five benchmark datasets. The experimental outcomes reveal that their proposed strategy outperformed existing methods on these datasets by a significant margin. However, their proposed system lacked the utilization of complementary information along with the integration of various features from both color and depth channels in order to create more state-of-the-art representations. Sharif et al. [18] proposed a hybrid technique for efficiently classifying daily human activities from an acquired video frame. In addition, their proposed system involves two significant steps. Initially, various subjects were detected in the acquired video frame via a combination of a new uniform and EM segmentation. Then, utilizing vector dimensions, they extracted local properties from specified sequences and combined them. Additionally, a new Euclidean distance along with joint entropy was exploited to pick the optimal features from the augmented vector. The optimal feature descriptors were catered to the classifiers for human activity recognition. However, occlusions were not addressed in this work. Another possibility is to incorporate saliency to maximize segmentation accuracy. In [19], Patel et al. proposed a method for detecting and recognizing the daily living activities of humans. Additionally, they explored various human visual databases to detect and monitor multiple human subjects. The background subtraction method was utilized to monitor the different persons in motion. In comparison, human daily living activities via the HOG feature extraction and an SVM classifier generate better recognition results with fewer false detections. Ji et al. [20] introduced a unique approach for interactive behavior recognition based on different stage probability fusions. Additionally, they dealt with the present issues in the interaction classification algorithms, including inadequate feature descriptors resulting from improper human body segmentation. Therefore, a multi-stage-based fusion strategy was presented to deal with this issue. However, this technique is ineffective at addressing the intrinsic characteristics of human behavior; instead, it is useful for categorizing abnormal behaviors, such as violent acts and unusual events. In [21], Wang et al. presented a probabilistic-based graphical framework for human physical activity recognition. Additionally, they addressed the issue of segmenting and recognizing continuous action. However, these methods operate only in offline mode. Ince et al. [22] developed a biometric system framework for detecting human physical activities in a three-dimensional space using skeletal joint angle patterns. Additionally, this framework exploits the RGB-depth camera, which appears suitable for video surveillance and elderly care facilities. However, there are a few drawbacks linked with the model. Initially, improper skeletal detection results in wrong angle estimations, and imprecise classifications.

### 2.2. HPAR via Wearable Sensors

Wearable-based inertial sensors have revolutionized every characteristic of our daily lives, from healthcare to ease and comfort. Therefore, due to the substantial demands for improved processing capacities and reduced size requirements, we analyzed IMU-based systems in this research. Irvine et al. [23] introduced a homogenous ensemble neural network method for identifying daily living activities in an indoor environment. Additionally, four standard models were developed and combined using support function fusion. Furthermore, they tested their proposed framework, the ensemble neural network method, by evaluating the attained HPAR performance with two non-parametric standard classifiers. The ensemble neural network technique outperformed both standard models, revealing the robustness of the proposed ensemble method. However, the work was restricted with no method for determining a relevant subset of input features. In [24], Feng et al. introduced an ensemble technique for recognizing HPAR, utilizing several wearable inertial sensors by integrating an independent random forest algorithm. The improved forecasting capabilities of the random forest resulted in a better option for wearable sensor-based healthcare tracking systems. Gupta et al. [25] presented an effective physical activity recognition system based on a portable wearable accelerometer that can be employed in a real-life application of elderly monitoring. Additionally, they incorporated effective capabilities for recognizing transitional behaviors. The proposed statistical features extracted additional information about the inertial signals in the time-frame window. Furthermore, additional cues are assessed to extract signal correlation. However, the fundamental challenge of this work is that only two individuals were used to acquire information, which limits the database’s applicability in various environments. In [26], Abidine et al. developed a weighted support vector machine (SVM) for tracking human life log activities in an indoor environment. Additionally, they addressed various implementation issues with the HAR methods, including redundant sequence characteristics and group variances in the learning set. To address these problems, they presented a novel technique for recognizing life log activities in an indoor environment. Furthermore, the entire model was based on the fusions of different algorithms, including PCA, SVM, and LDA. To begin, the learning set was lessened via the PCA and LDA features. Then, an SVM classifier was used for each class to handle the unbalanced life log activity database to maximize the detection rate. In another study, Cillis et al. [27] proposed a ubiquitous novel solution for locomotion patterns via a wearable-based inertial accelerometer sensor. Additionally, their proposed model utilized a finite feature set along with a decision tree classifier to recognize four distinct human locomotion patterns. Firstly, they acquired features from both individual and dynamic sets of windows. The experimental outcomes indicated that accuracy was better when performing static tasks but much lower when performing dynamic tasks. The model’s low processing overhead may make it well-suited for real-time applications in medical care. In [28], Tian et al. presented an ensemble learning approach for recognizing human physical activities. Three state-of-the-art classifiers and multiple SVMs were trained by numerous features, resulting in an ensemble learning-based system. Additionally, an adaptive hybrid model extracted various features from human physical activities to improve their recognition rate. Jing et al. [29] developed a HAR-based system for tracking life log daily activities along with fall detection by using various wearable inertial sensors. Javed et al. [30] presented a state-of-the-art technique to recognize human physical activities via sensory data acquired from a two-axis smartphone accelerometer. In addition, this study also determined the efficacy and impact of the individual accelerometer axis in classifying human physical activities. Furthermore, this technique incorporates multi-modal sensory data acquired from three body-worn sensors. This study demonstrates that the augmentation of inertial sensor data improves the HAR accuracy. The entire system was compared to a complete activity set comprising cyclic, static, and random actions. Furthermore, time and frequency domain features were extracted to gain optimal results.

## 3. Material and Methods

**Figure 1:**
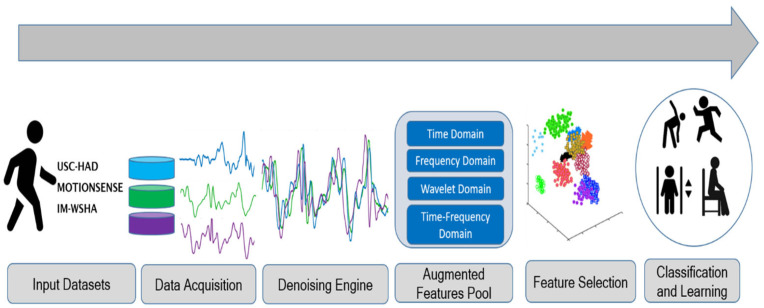
The proposed architecture of the HPAR system.

The proposed HPAR system acquired raw signals from five benchmark datasets comprising MEMS inertial sensors. To begin, a preprocessing step was employed to eliminate saw-tooth wave noise caused by abrupt displacement using a third-order median filter. Next, the filtered signal values were organized into time blocks of comparable duration. Secondly, in feature extraction, we proposed an augmented features pool comprising five different features in four domains: time, frequency, wavelet, and time–frequency domain. Additionally, the acquired features were normalized using extreme values to eliminate the possibility of complex values appearing during the final phases of feature selection. Thirdly, a feature selection strategy was adopted for optimizing feature vectors in such a way that the relevant optimal features were retained for further phases of data processing. Finally, the denoised optimal selected features were served to the random forest classifier algorithm, which analyzed the signal stream and trained and tested the model via the optimal feature descriptor set. The proposed architecture of HPAR is presented in Figure 1.

### 3.1. Data Acquisition and Signal Denoising

**Figure 2:**
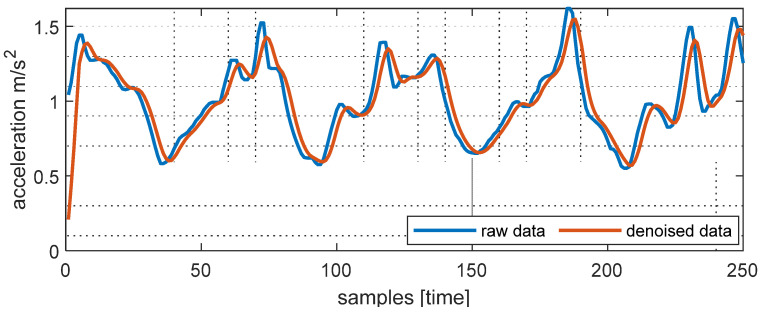
Signal denoising for the wearable inertial signals in the proposed HPAR framework.

Feature extraction was highly dependent on the denoising stage, so it was critical to remove all noise from the acquired raw data [31]. The data collected from the sensors comprising the inertial measurement unit and MEMS were seriously vulnerable to interference and noise, resulting in raw signal variances and, consequently, feature loss. As a result, we utilized a median filter for inertial sensor-based benchmark datasets to reduce the related noise. The denoised and unprocessed signal components of the third-order median filter of the inertial sensor are illustrated in Figure 2.

### 3.2. Feature Extraction

In this phase, we proposed an augmented features model to obtain important feature descriptors to assist the analysis of inertial-based signals. Additionally, it was composed of four different major domains—time, frequency, wavelet, and frequency domain descriptors. The filtered signals were streamed and used to abstract features from the sensor data stream. Furthermore, signal features were retrieved from within the confined region with adequate contextual information.

#### 3.2.1. Statistical Features

**Figure 3:**
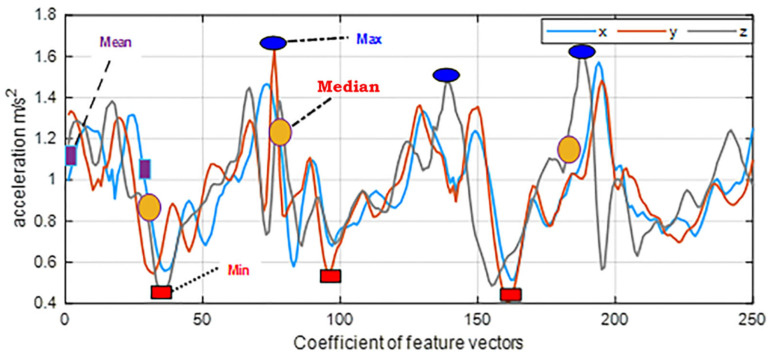
Three-axis vector plot of statistical features of daily life log activity (walking) from the USC-HAD dataset.

The statistical descriptors (S_d_) depict the average mean, mode, median, and min/max signal features of the IMU signal. Additionally, these descriptors are important in assessing the aggregate differences that come from each *n* physical activity.

(1) $$\mathrm{Sd}=\sum_{c=1}^{n}\frac{a_{r}}{n},\frac{\sum_{c=1}^{n}{\left(I-\;\bar{I}\right)}^{2}}{n-1},mi\left(\mathrm{signal}\right)\left(M_{i}\right),mx\left(\mathrm{signal}\right)\left(X_{i}\right)\;$$

where *n* is the framed vector data size, *a* is the whole number of coefficients in the vector, V depicts the initial vector value, and $\;\bar{I}$ represents the average mean of the vector data. Figure 3 showed a three-axis plot augmented with different time domain features of walking activities extracted from the MOTIONSENSE dataset.

#### 3.2.2. Hilbert–Huang Transform (HHT)

**Figure 4:**
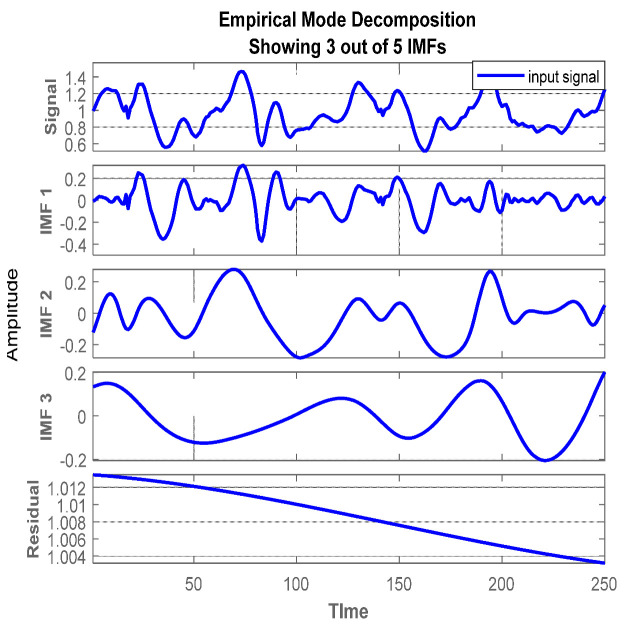
The sifting process of the empirical mode decomposition of inertial components from the USC−HAD dataset. From top to bottom, an input signal and intrinsic mode functions 1, 2, and 3. Finally, IMF is reduced from the input.

The HHT is believed to be highly effective for dealing with non-linear and stochastic signal data [32]. For instance, data from five benchmark datasets involved different inertial time series data. Additionally, the IMU data from different sensors were generally non-linear. Thus, the Hilbert–Huang transform (HHT) divided the resultant time series of non-linear IMU data into distinctive repeated components called intrinsic mode functions (IMFs) (see Figure 4). The whole method is known as the intrinsic mode decomposition. Additionally, these elements generated distinct frequency bands capable of computing shifts in instantaneous frequencies. Therefore, we could make valid comparisons between the attributes of diverse activities. The acquired processed data can be expressed as:

(2) $$P\left(s\right)=\overset{n}{\underset{a=1}{\sum }}c_{a}+r_{n}$$

where *P*(*s*) represents the processed inertial signal, c_a_ indicates the *a*th IMF, and $r_{n}$ depicts the whole remainder.

#### 3.2.3. Haar Wavelet Transform

**Figure 5:**
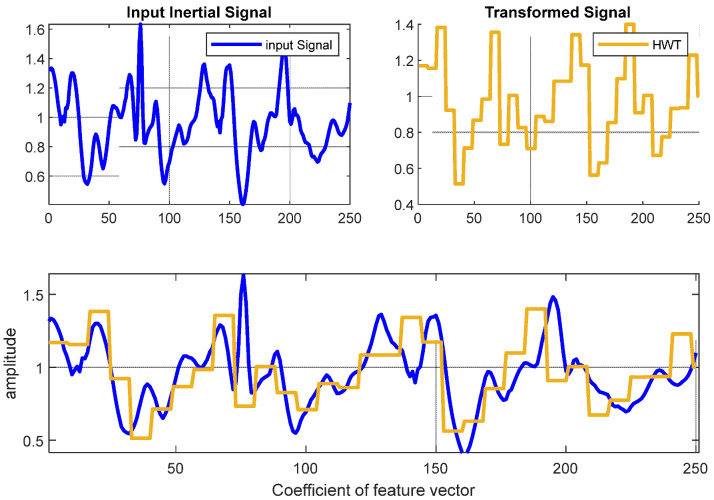
The 1D-HWT feature of the inertial signal feature plot from daily activity (walking) from the USC-HAD dataset.

The Haar wavelet transform (HWT) has evolved as a sophisticated technology in the domain of image and signal analysis. In general, wavelets are mathematical techniques utilized for hierarchically splitting functions [33]. In our HPAR model, the Haar wavelet-based features were used to recognize patterns at specific intervals in order to examine signal variations. In addition, the Haar wavelet transform involves a wavelet-based structure (see Figure 5). Therefore, it is a robust and reliable signal processing technique. HWTs are denoted by their coefficients (a, d), with ‘a’ depicting approximation coefficients and ‘d’ representing the approximation coefficients. Moreover, these coefficients facilitate estimating the IMU signal’s total power and serve inappropriate restoration and segmentation. The HWT can be expressed as:

(3) $$\psi \left(f\right)=\{\begin{array}{cc} 1 & 0\leq a\leq \frac{1}{2}\\ -1 & \frac{1}{2}\leq a\leq 1\\ 0 & \;\mathrm{else}\; \end{array}$$

where the scaling function is expressed as ψ(f).

#### 3.2.4. Spectral Entropy

**Figure 6:**
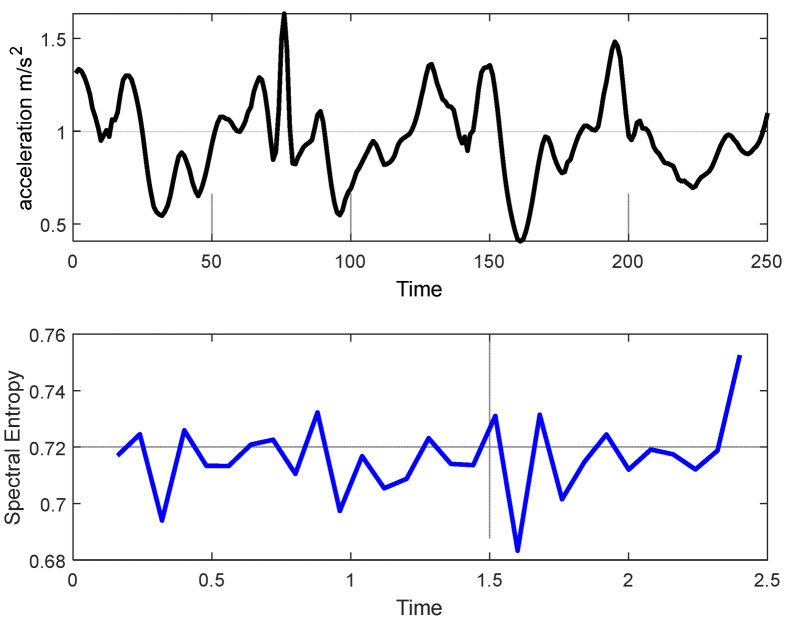
Spectral entropy for the upstairs walking activity signal plot from the MOTIONSENSE dataset. The black signal denotes inertial data and the blue signal represents the spectral entropy of an inertial signal from the MOTIONSENSE dataset.

Spectral entropy quantifies the randomness in a model, which contributes to the system’s complexity [34]. The system’s complexity provides significant information, such as random variations in body activity. These data are utilized to distinguish between various life log activities (see Figure 6). Additionally, they assist in estimating the IMU signal spectral range, which generates a power spectrum involving important information about a particular activity. The following steps were used to acquire the features presented by spectral entropy.

- Firstly, the acquired IMU signal’s power spectrum was normalized and denoted as *Ps_p_(f)*.

(4) $$Q_{sp}\left(f\right)=\frac{P_{sp}\left(f\right)}{\sum_{f}P_{sp}\left(f\right)}\;$$

- To extract modified elements, we utilized the Shannon function to change the normalized power spectrum.

(5) $$T_{sp}\left(f\right)=Q_{sp}\left(f\right)log\frac{1}{Q_{sp}\left(f\right)}\;$$

- In the end, the acquired $Q_{sp}\left(f\right)$ elements were enveloped.
  (6) $${SE\;}_{sp}=\frac{\sum_{f}Q_{sp}\left(f_{i}\right)}{\log\left(N_{sp}\left(f_{1},f_{2}\right)\right)}\;$$
  where *SE_sp_* is equivalent to the number of elements in total.

#### 3.2.5. Wavelet Packet Entropy (WPE)

**Figure 7:**
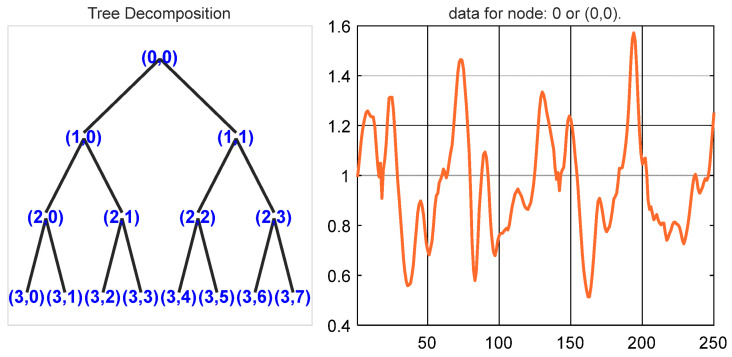
Wavelet packet entropy (two-level) decomposition for the inertial data (for walking data from the USC-HAD dataset).

Wavelet packet entropy is a time–frequency representation technique that is both effective and reliable for inertial signals. Initially, WPE decomposes an inertial signal into many frequency resolutions, each with its own set of information and approximation factors [35]. The two-level decomposition of walking data is presented in Figure 7. Additionally, WPE can be represented as:

(7) $$d_{pe}=\{\begin{array}{l} d_{0,0}\left(a\right)=p\left(a\right)\\ d_{i,2j-1}\left(r\right)=\sqrt{2}\underset{c}{\sum }h\left(c\right)d_{i-1,j}\left(2r-c\right)\\ d_{l,2j}\left(r\right)=\sqrt{2}\underset{c}{\sum }g\left(c\right)d_{i-1,j}\left(2r-c\right) \end{array}$$

where *h*(*c*) along with *g*(*c*) denotes two different filters for the extraction of ACs and DCs, and *d_i,j_* indicates the restoration of IMU signals at the *i*th and *j*th node.

### 3.3. Feature Selection via Stochastic Gradient Descent (SGD)

In the proposed HPAR model, features from the different domains were optimized using a state-of-the-art gradient algorithm, referred to as a stochastic gradient algorithm. Gradient descent is an important method for discovering the optimal solution with the lowest cost function via a linear function. Initially, gradient descent was utilized to adapt network gradients in neural networks [36]. Additionally, the gradient descent approach may work slower if all the training data are evaluated at each epoch. Furthermore, in some cases, SGD outperforms the other gradient optimizers, such as Adam, in terms of adaptability to new data [37]. The training phase ends when the loss on the validation set exceeds the threshold level. Due to the fact that SGD generates more oscillations throughout the training phase, it requires a more significant number of epochs to converge. Considering the extended training period, SGD has two significant advantages. To begin with, the stochastic technique improves the probability of outperforming local minima solutions [38,39]. Then this lowers the risk of abruptly interrupting the training process by ensuring that the model has been through a sufficient number of epochs [40,41]. Therefore, we present the SGD approach with the minibatch as a non-consumptive optimizer. However, when incorporated with sparse data selection, the minibatch SGD significantly lowers the cost and inconsistency associated with the traditional SGD. Thus, the minibatch involves a comprehensive analysis combined with adaptive learning rates and initial settings to attain the minimum loss function. As a result, the learning settings are adjusted, and the result is attained reliant on the learning rate. Thus, the first learning rate was set to default 0.01, and the average batch size was set to 1000, which may be tuned via regularization parameters. The SGD model for the entire training sets for *i*(*k*)and *j*(*k*) is as follows:

(8) $$\theta =\theta -\eta \cdot \nabla_{\theta }J\left(\theta ;i^{\left(r\right)};j^{\left(r\right)}\right)$$

where θ shows the main angle, $\nabla_{\theta }J\left(\theta ;i^{\left(r\right)};j^{\left(r\right)}\right)$ are the main functions, and η signifies the size of the minibatch, and the lowest loss function is denoted by:

(9) $$\theta =\theta -\eta \cdot \nabla_{\theta }J\left(\theta ;i^{\left(r:r+n_{bs}\right)};j^{\left(r:r+n_{bs}\right)}\right)$$

where θ shows the angle and $\nabla_{\theta }J\left(\theta ;i^{\left(r:r+n_{bs}\right)};j^{\left(r:r+n_{bs}\right)}\right)$ the updated main function.

### 3.4. Classification

**Figure 8:**
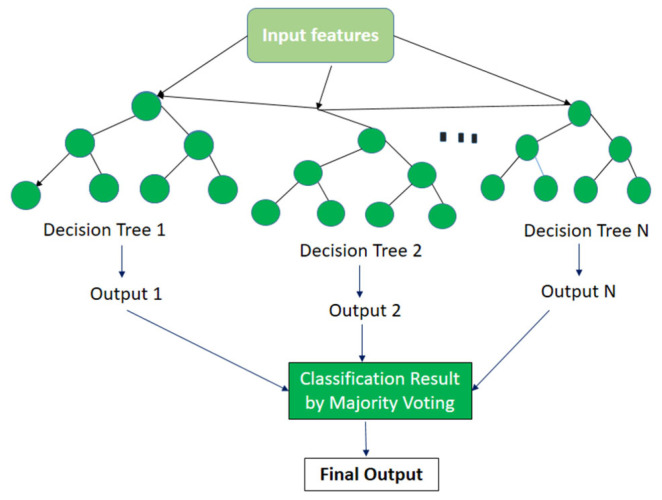
Proposed HPAR model from the random forest classifier.

**Figure 9:**
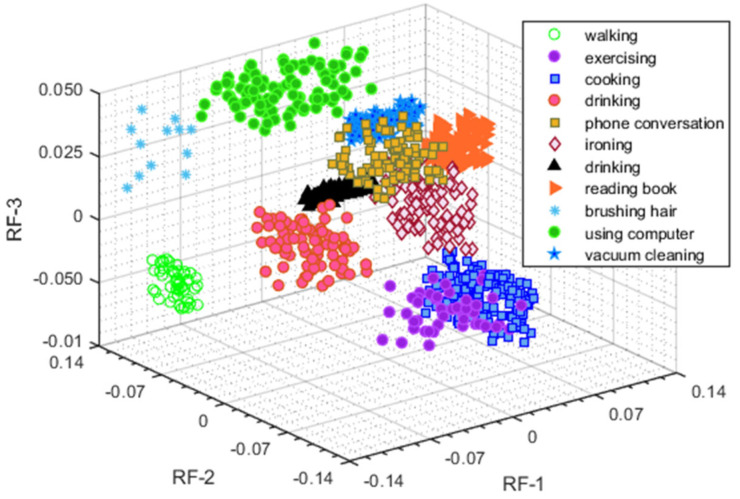
Random forest recognition on the IM−WSHA benchmark dataset.

After the feature selection step, we tested our proposed HPAR model from five benchmark datasets, IM-WSHA, PAMAP-2, UCI HAR, MobiAct, and MOTIONSENSE, which were composed of diverse classes of human daily living activities. The optimal feature descriptors of SGD were recognized by a state-of-the-art classifier, random forest (RF), which followed ensemble learning techniques for classification and regression. Additionally, the random forest classifier included a novel variant of bagged trees, which is an optimal method for creating a training test. In our case, bagging acquired samples from all five daily living activities datasets. A model was built for each sample and was utilized to make decision trees. Finally, all decision trees were augmented based on the highest number of votes to deliver the best results. Figure 8 illustrates the overall architecture of the random forest classifier. The classified vectors for the IM-WSHA dataset are shown in Figure 9.

For *r* = 1, … *R*: Samples, with an alternative, *n* training examples from *A*, *B*, are also referred to as *A_r_*, *B_r_*.

We trained a proposed model, *f_r_* on *A_r_*, *B_r_*.

(10) $$\hat{f}=\frac{1}{R}\overset{R}{\underset{r=1}{\sum }}f_{r}\left(y^{\prime }\right)$$

where *y′* indicates the predictions for the random samples. It was calculated by averaging the prediction of all decision trees on *y′*. The total number of samples is represented as *R*, which is a free parameter.

## 4. Discussion

All experiments and testing were performed using an HP laptop configured with an Intel Core i5-8300H CPU operating at a base frequency of 2.30 GHz, 8GB RAM, and Nvidia GTX 1050Ti dedicated graphics card running Windows 10 Pro 64-bit with Google Colab and MATLAB. Additionally, a model for evaluating the performance of our HPAR system from five benchmark datasets was constructed. Furthermore, we used the leave-one-subject-out (LOSO) cross-validation scheme to assess the recognition performance of our HPAR model in different indoor and outdoor settings.

### 4.1. Benchmark Datasets

The first benchmark dataset—the IM-Wearable Smart Home Activities (IM-WSHA) [42] database—contains signal data from five IMU sensors, including three-axis accelerometers, gyroscopes, and magnetometers. Additionally, these IMU sensors were incorporated into three separate bodily regions, the chest, thigh, and wrist, to extract real-time human motion features of daily living activities. Ten individuals (five males and five females) attempted eleven different physical activities in the indoor setting, including walking, exercising, cooking, drinking, phone conversation, ironing, watching TV, reading a book, brushing hair, using the computer, and vacuum-cleaning.

The second benchmark dataset—physical activity monitoring for aging people, also referred to as the PAMAP-2 [43] dataset—is openly accessible via the UCI learning repository. The PAMAP-2 database involved data from three wireless inertial sensors incorporated with three-axis accelerometers, gyros, and magnetometers that were worn on the individual’s wrist, chest, and ankle positions during 18 daily physical static and dynamic activities. However, this dataset evaluated twelve living activities, including walking, cycling, lying down, sitting, standing, Nordic walking, running, rope jumping, ironing, house cleaning, and ascending and descending stairs. Furthermore, this database involved recurring daily activities unique to the HPAR model to analyze the sophisticated motion patterns.

The third benchmark dataset—the MOTIONSENSE [44] dataset—is a publicly available open-access database that involves smartphone tri-axial accelerometers and tri-axial gyroscope sensor data. The human subject placed his smartphone in his front pocket. A total of 24 individuals (14 males and 10 females) performed six life log activities in both indoor and outdoor settings (such as walking, sitting, running, standing, ascending, and descending activities).

The fourth benchmark dataset was the Human Activity Recognition database (UCI HAR) [45]. Researchers acquired triaxial linear acceleration and rotational motion data using the cellphone accelerometer sensor at a data rate of 50 Hz. Such data were normalized for denoising with a median filter and a low Butterworth filter with a 20 Hz sample rate. This frequency is appropriate for detecting human body movements since 99% of its potential is confined to 15 Hz. The speed information, which comprises gravitational and body motion characteristics, was split using each Butterworth low-pass filtration system as body acceleration and gravity.

The fifth benchmark dataset, the MobiAct dataset [46], consists of tri-axial data for 15 activities of daily living (ADLs) and falls from 67 individuals, captured using a Samsung Galaxy S3. Designers examined a frame size of 5 s with a sampling frequency of 87 Hz. Moreover, the individual’s sex, age, body weight, and size were mentioned. The device was randomly oriented within a flexible area selected by the individual. The sampling frequency was originally 87 Hz. Table 1 presents a comprehensive comparison of the five benchmark datasets.

### 4.2. Experimental Result and Evaluation

**Figure 10:**
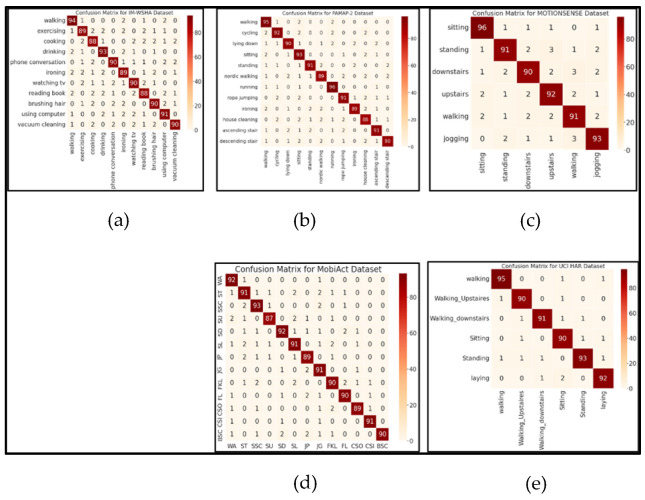
Confusion matrices of (**a**) the 11 daily living activities on the IM-WSHA dataset via random forest, (**b**) 12 physical activities on the PAMAP-2 dataset via random forest, (**c**) 6 locomotion activities on the MOTIONSENSE dataset via random forest, (**d**) 6 locomotion activities on the UCI HAR dataset via random forest, (**e**) 6 locomotion activities on the MobiAct dataset via random forest. WA = walking, ST = standing, SSC = stand to sit on chair, SU = stairs up. SD = stairs down, SL = sideward-laying, JP = jumping, JG = jogging, FKL= front-knees-laying, FL = forward-laying, CSO = car-step-out, CSI = car-step in, BSC = back-sitting-chair.

We evaluated the performance of a state-of-the-art random forest classifier by catering to the optimal selected features of different domains, including statistical, HHT, HWT, spectral entropy, and wavelet packet entropy descriptors via the PAMAP-2, MOTIONSENSE, UC HAR, MobiAct, and IM-WASHA benchmark databases. The experimental evaluation was conducted three-fold to assess the performance of the HPAR framework from three benchmark datasets. Figure 10a presents the confusion matrix for the IM-WSHA dataset for eleven daily living activities, where 90.18% of total accuracy was achieved. In the PAMAP-2 dataset, Figure 10b indicates a recognition rate of 91.25% from twelve physical activities. Regarding the MOTIONSENSE dataset, Figure 10c depicts an average accuracy of 92.16% from six static and dynamic activities, including walking, sitting, standing, jogging, upstairs, and downstairs. On the other hand, smartphone-based inertial sensor datasets, namely UCI-HAR and MobiAct, achieved significant results. Figure 10d shows that the confusion matrix UCI HAR of the dataset attained a significant mean accuracy of 91.83%. Figure 10e presents the confusion matrix of the MobiAct dataset, which achieved a 90.46% recognition rate.

**Figure 11:**
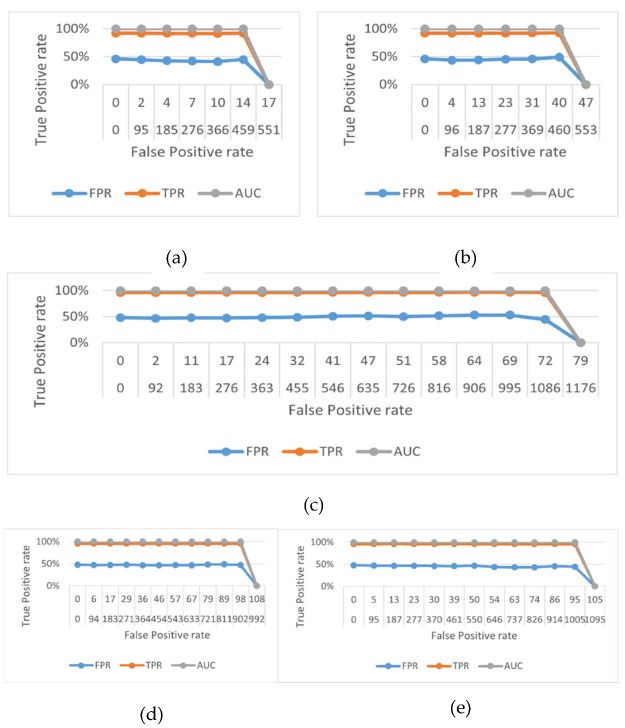
Receiver operating characteristic (ROC) curve on the (**a**) UCI HAR dataset, (**b**) MOTIONSENSE dataset, (**c**) MobiAct dataset, (**d**) PAMAP-2 dataset, (**e**) IMWSHA dataset.

Figure 11a–e shows the receiver operating characteristic (ROC) curve of six locomotion activities on the UCI HAR, MOTIONSENSE, MobiAct, PAMAP-2, and IMWSHA datasets.

In Table 2, Table 3, Table 4, Table 5 and Table 6, we present the HPAR system performance with two state-of-the-art techniques, the support vector machine (SVM) [47] and AdaBoost [48] classifiers, using accuracy and other performance metrics, such as accuracy, recall, precision, and F measures for all activity classes in five databases.

Similarly, in Table 7, we provide the Cohen’s kappa and Matthews correlation coefficient from all datasets. Finally, in Table 8, we summarize the results of the comparison between the HPAR model and different state-of-the-art systems. 

## 5. Conclusions

In this study, we presented an HPAR system based on augmented feature descriptors, comprising four major domain features. These domains analyzed statistical descriptors, the Hilbert–Huang transform, the Haar wavelet transform, spectral entropy, and wavelet packet entropy descriptors. Additionally, these augmented-based descriptors optimized the performance of the proposed HPAR systems by assessing spatiotemporal moments and continuous motion patterns of human daily living activities. Furthermore, these descriptors were optimized via stochastic gradient descent (SGD) and were then catered to the random forest (RF) classifier for further classification. This work also compares the performance of the SGD-based random forest classifier with other state-of-the-art classifiers, such as support vector machine (SVM) and AdaBoost. Our system incorporates data processing methods, robust feature extraction methods, and classification algorithms that have the potential to outperform the other state-of-the-art recognition rates.

In future studies, we will employ more sophisticated activities and behaviors from various contexts, including healthcare units, sports centers, and smart home environments, via different inertial sensors. Additionally, we intend to develop self-annotated datasets for smart healthcare using multi-modal sensors.

## Figures and Tables

**Table 1:** A detailed comparison of our self-annotated IM-WSHA dataset and other benchmark datasets with sample sizes, other similarities, and differences.

Dataset	Sensors	Sample Rate	Activities	Subjects
IM-WSHA (Self-annotated)	3-IMUs	100 Hz	Cooking, drinking, reading a book, walking, etc.	10
PAMAP-2	3-IMUs	9 Hz	Sitting, standing, walking, ironing, cycling, etc.	9
MobiAct	Smartphone	20 Hz	Standing, walking, jogging, lying	19
UCI-HAR	Accelerometer and gyroscope	50 Hz	Walking, walking upstairs, walking downstairs	30
MOTIONSENSE	Smartphone	50 Hz	walking Jogging, downstairs, upstairs, etc.	24

**Table 2:** Comparison of the evaluation metrics (precision, recall, and F1 score) of the HPAR model from the IM-WSHA dataset.

Methods	Random Forest			SVM-RBF			AdaBoost		
Activities	Precision	Recall	F1 Score	Precision	Recall	F1 Score	Precision	Recall	F1 Score
**W1**	0.912	0.940	0.926	0.883	0.885	0.879	0.831	0.824	0.827
**W2**	0.898	0.890	0.894	0.875	0.868	0.864	0.827	0.815	0.820
**W3**	0.880	0.880	0.880	0.868	0.865	0.882	0.839	0.828	0.833
**W4**	0.902	0.930	0.916	0.841	0.854	0.857	0.815	0.812	0.813
**W5**	0.918	0.900	0.909	0.909	0.903	0.911	0.846	0.831	0.838
**W6**	0.881	0.880	0.885	0.872	0.870	0.880	0.844	0.837	0.840
**W7**	0.900	0.900	0.900	0.881	0.871	0.868	0.831	0.825	0.827
**Activities**	**Precision**	**Recall**	**F1 Score**	**Precision**	**Recall**	**F1 Score**	**Precision**	**Recall**	**F1 Score**
**W8**	0.888	0.910	0.884	0.870	0.869	0.867	0.824	0.816	0.819
**W9**	0.909	0.900	0.904	0.881	0.877	0.881	0.853	0.845	0.848
**W10**	0.900	0.910	0.905	0.882	0.879	0.877	0.829	0.819	0.823
**W11**	0.927	0.900	0.913	0.895	0.892	0.900	0.846	0.837	0.841
**Mean**	**0.901**	**0.903**	**0.901**	**0.878**	**0.875**	**0.878**	**0.835**	**0.826**	**0.829**

W1 = walking; W2 = exercising; W3 = cooking; W4 = drinking; W5 = phone conversation; W6 = ironing; W7 = watching tv; W8 = reading book; W9 = brushing hair; W10 = using computer; W11 = vacuum cleaning.

**Table 3:** Comparison of the evaluation metrics (precision, recall, and F1 score) of the HPAR model from the PAMAP-2 dataset.

Methods	Random Forest			SVM-RBF			AdaBoost		
Activities	Precision	Recall	F1 Score	Precision	Recall	F1 Score	Precision	Recall	F1 Score
**A1**	0.887	0.950	0.917	0.884	0.875	0.884	0.837	0.824	0.830
**A2**	0.920	0.920	0.920	0.861	0.864	0.861	0.833	0.826	0.829
**A3**	0.927	0.940	0.933	0.894	0.873	0.894	0.841	0.883	0.861
**A4**	0.861	0.900	0.880	0.849	0.871	0.849	0.815	0.820	0.817
**A5**	0.938	0.920	0.928	0.914	0.917	0.914	0.841	0.830	0.835
**A6**	0.908	0.910	0.909	0.897	0.884	0.897	0.838	0.829	0.833
**A7**	0.923	0.930	0.926	0.875	0.861	0.875	0.829	0.834	0.831
**A8**	0.910	0.880	0.894	0.867	0.863	0.867	0.821	0.817	0.818
**A9**	0.946	0.920	0.933	0.877	0.881	0.877	0.831	0.827	0.828
**A10**	0.880	0.910	0.894	0.836	0.832	0.836	0.836	0.827	0.831
**A11**	0.919	0.890	0.904	0.896	0.909	0.896	0.842	0.831	0.836
**A12**	0.937	0.930	0.933	0.892	0.903	0.892	0.846	0.838	0.841
**Mean**	**0.913**	**0.916**	**0.914**	**0.878**	**0.877**	**0.878**	**0.834**	**0.832**	**0.833**

A1 = walking; A2 = cycling; A3 = lying down; A4 = sitting; A5 = standing; A6 = nordic walking; A7 = running; A8 = rope jumping; A9 = ironing; A10 = house cleaning; A11 = ascending stair; A12 = descending stair.

**Table 4:** Comparison of the evaluation metrics (precision, recall, and F1 score) of the HPAR model from the MOTIONSENSE dataset.

Methods	Random Forest			SVM-RBF			AdaBoost		
Activities	Precision	Recall	F1 Score	Precision	Recall	F1 Score	Precision	Recall	F1 Score
**M1**	0.941	0.960	0.950	0.885	0.888	0.886	0.788	0.796	0.791
**M2**	0.928	0.910	0.919	0.901	0.882	0.891	0.825	0.795	0.809
**M3**	0.918	0.900	0.909	0.791	0.810	0.800	0.768	0.790	0.778
**M4**	0.911	0.920	0.915	0.769	0.750	0.759	0.745	0.702	0.722
**M5**	0.910	0.910	0.910	0.759	0.766	0.762	0.736	0.737	0.736
**M6**	0.920	0.930	0.925	0.785	0.783	0.783	0.751	0.712	0.730
**Mean**	**0.921**	**0.922**	**0.921**	**0.815**	**0.813**	**0.814**	**0.768**	**0.755**	**0.761**

M1 = sitting; M2 = standing; M3 = downstairs; M4 = upstairs; M5 = walking; M6 = jogging.

**Table 5:** Comparison of the evaluation metrics (precision, recall, and F1 score) of the HPAR model from the UCI-HAR dataset.

Methods	Random Forest			AdaBoost			SVM-RBF		
Activities	Precision	Recall	F1 Score	Precision	Recall	F1 Score	Precision	Recall	F1 Score
**U1**	0.979	0.979	0.979	0.978	0.957	0.968	0.976	0.976	0.976
**U2**	0.968	0.978	0.973	0.967	0.978	0.973	0.976	0.964	0.970
**U3**	0.978	0.968	0.973	0.977	0.977	0.977	0.963	1.000	0.981
**U4**	0.947	0.968	0.957	0.943	0.965	0.954	0.974	0.974	0.974
**U5**	0.979	0.959	0.969	0.988	0.977	0.983	0.975	0.963	0.969
**U6**	0.968	0.968	0.968	0.967	0.967	0.967	0.976	0.964	0.970
**Mean**	**0.970**	**0.970**	**0.970**	**0.970**	**0.970**	**0.970**	**0.973**	**0.974**	**0.973**

U1 = walking; U2 = walking upstairs; U3 = walking downstairs; U4 = sitting; U5 = standing; U6 = laying.

**Table 6:** Comparison of the evaluation metrics (precision, recall, and F1 score) of the HPAR model from the MobiAct dataset.

Methods	Random Forest			AdaBoost			SVM-RBF		
Activities	Precision	Recall	F1 Score	Precision	Recall	F1 Score	Precision	Recall	F1 Score
**B1**	0.920	0.979	0.948	0.919	0.978	0.948	0.917	0.978	0.946
**B2**	0.901	0.910	0.905	0.943	0.892	0.917	0.935	0.946	0.941
**B3**	0.959	0.939	0.949	0.933	0.944	0.939	0.955	0.955	0.955
**B4**	0.956	0.926	0.941	0.956	0.916	0.935	0.955	0.944	0.949
**B5**	0.948	0.920	0.934	0.969	0.949	0.959	0.965	0.912	0.938
**B6**	0.919	0.910	0.915	0.936	0.957	0.946	0.976	0.922	0.949
**B7**	0.918	0.937	0.927	0.934	0.934	0.934	0.976	0.953	0.964
**B8**	0.892	0.958	0.924	0.887	0.956	0.920	0.920	0.988	0.952
**B9**	0.918	0.928	0.923	0.957	0.967	0.962	0.908	0.963	0.935
**B10**	0.938	0.938	0.938	0.977	0.945	0.961	0.952	0.952	0.952
**B11**	0.957	0.947	0.952	0.976	0.953	0.964	0.964	0.942	0.953
**B12**	0.968	0.968	0.968	0.964	0.964	0.964	0.964	0.964	0.964
**B13**	1.000	0.928	0.963	0.953	0.943	0.948	1.000	0.965	0.982
**Mean**	**0.938**	**0.937**	**0.937**	**0.947**	**0.946**	**0.946**	**0.953**	**0.953**	**0.952**

B1 = walking; B2 = standing; B3 = stand to sit on chair; B4 = stairs up; B5 = stairs down; B6 = sideward-laying; B7 = jumping; B8 = jogging; B9 = front-knees-laying; B10 = forward-laying; B11 = car-step-out; B12 = car-step-out; B13 = back-sitting-chair.

**Table 7:** Cohen’s kappa and Matthews correlation coefficient from all datasets.

Activities	MOTIONSENSE	IM-WSHA	PAMAP-2	MobiAct	UCI-HR
Mean MCC value	0.90	0.89	0.90	0.93	0.96

**Table 8:** Comparison of the recognition rate of the proposed HPAR model with other state-of-the-art methods from the IM-WSHA, PAMAP-2, UCI-HR, MobiAct, and MOTIONSENSE datasets.

Methods	MOTIONSENSE (%)	PAMAP-2 (%)	IM-WSHA (%)	UCI-HR (%)	MobiAct (%)
Bidirectional LSTM [49]	-	64.10	-	-	-
AdaBoost [50]	-	77.78	81.30	-	-
BERT model [51]	79.86	-	-	-	-
Deep convolutional network [52]	-	87.50	-	-	-
Kinematics features and kernel sliding perceptron [53]	-	90.49	84.50	-	-
Ensemble learning [54]	-	90.11	-	-	-
Multi-fused features [55]	88.25	-	-	-	-
KNN classification [56]	-	-	75.30	-	-
Optimized method [57]	87.50	-	-	-	-
Actionlet ensemble [58]	-	-	-	88.20	-
COV-JH-SVM [59]	-	-	-	80.40	-
FTP-SVM [60]	-	-	-	90.01	-
Threshold technique [61]	-	-	-	-	81.30
SVM [62]	-	-	-	-	77.93
CNN [63]	-	-	-	-	80.71
Coupled GRU [64]	-	-	-	88.50	-
SSMN [65]	-	-	-	81.00	87.90
Proposed HPAR System	**92.16**	**91.25**	**90.18**	**91.83**	**90.46**

Bold letters for proposed HPAR system recognition of all datasets.

## Data Availability

https://www.researchgate.net/publication/343812965_Intelligent_Media_-_Wearable_Smart_Home_Activities_IM-WSHA_Dataset (accessed on 20 May 2022). https://github.com/SheikhBadaruddinTahir/IM-WSHA) (accessed on 27 June 2022). https://archive.ics.uci.edu/ml/datasets/PAMAP2+Physical+Activity+Monitoring (accessed on 3 July 2022). https://www.kaggle.com/malekzadeh/motionsense-dataset (accessed on 20 June 2022). https://archive.ics.uci.edu/ml/datasets/human+activity+recognition+using+smartphones (accessed on 3 July 2022). https://bmi.hmu.gr/the-mobifall-and-mobiact-datasets-2/ (accessed on 4 July 2022).
